# *Staphylococcus aureus* SOS response: Activation, impact, and drug targets

**DOI:** 10.1002/mlf2.12137

**Published:** 2024-09-30

**Authors:** Kaiying Cheng, Yukang Sun, Huan Yu, Yingxuan Hu, Yini He, Yuanyuan Shen

**Affiliations:** ^1^ Zhejiang Key Laboratory of Medical Epigenetics, Department of Immunology and Pathogen Biology, School of Basic Medical Sciences, Affiliated Hospital of Hangzhou Normal University Hangzhou Normal University Hangzhou China; ^2^ State Key Laboratory for Diagnosis and Treatment of Infectious Diseases, The First Affiliated Hospital, College of Medicine Zhejiang University Hangzhou China

**Keywords:** antibiotic‐resistant, drug target, error‐prone repair, SOS response, *Staphylococcus*

## Abstract

*Staphylococcus aureus* is a common cause of diverse infections, ranging from superficial to invasive, affecting both humans and animals. The widespread use of antibiotics in clinical treatments has led to the emergence of antibiotic‐resistant strains and small colony variants. This surge presents a significant challenge in eliminating infections and undermines the efficacy of available treatments. The bacterial Save Our Souls (SOS) response, triggered by genotoxic stressors, encompasses host immune defenses and antibiotics, playing a crucial role in bacterial survival, invasiveness, virulence, and drug resistance. Accumulating evidence underscores the pivotal role of the SOS response system in the pathogenicity of *S. aureus*. Inhibiting this system offers a promising approach for effective bactericidal treatments and curbing the evolution of antimicrobial resistance. Here, we provide a comprehensive review of the activation, impact, and key proteins associated with the SOS response in *S. aureus*. Additionally, perspectives on therapeutic strategies targeting the SOS response for *S. aureus*, both individually and in combination with traditional antibiotics are proposed.

## INTRODUCTION

When pathogens invade the host organism, the host's immune response triggers the production of reactive oxygen species (ROS), which cause double‐strand DNA breaks (DSBs) in the bacterial genome, ultimately resulting in bacterial cell death^1^. Quinolone antibiotics are capable of inducing DNA topoisomerase‐dependent DSBs, thus aiding in bacterial eradication^2^. However, bacteria can persist and thrive by effectively repairing the aforementioned DSBs if their DNA repair system is robust.

The Save Our Souls (SOS) response is a cellular mechanism employed by bacteria, serving as an emergency system to manage and repair DNA lesions caused by various stressors. It involves a complex transcriptional reaction to DNA damage, resulting in cell cycle arrest and the initiation of the DNA repair process. This response is regulated by two pivotal proteins: the LexA transcriptional repressor, responsible for repressing the SOS genes under normal conditions, and the SOS response inducer RecA protein, which regulates the autocleavage of LexA and activates the transcription of SOS genes^3^, ^4^. The SOS response can introduce numerous mutations while preserving the integrity of the pathogen's genome. This response also triggers the dissemination of pathogenicity islands and resistance gene cassettes, further exacerbating the formation of drug‐resistant strains^5^, ^6^. Moreover, this system is implicated in quinolone antibiotics inducing pathogens to produce small colony variants (SCVs), leading to severe chronic infections and posing a significant challenge in clinical treatment^7^. Hence, the pathogen's SOS response system significantly undermines the antibacterial effects of the host's immune response and antibiotics.

The rapid surge of antibiotic resistance in bacterial pathogens is now recognized as a major global health crisis^8^. Novel strategies are required to halt the development of resistance and enhance the effectiveness of antibiotics. The SOS response represents a promising target for developing therapeutics aimed at reducing the acquisition of antibiotic resistance and enhancing the bactericidal activity of antimicrobial agents, such as quinolones. Screening or designing drugs that target key proteins within the pathogen's SOS response system is anticipated to weaken DNA repair capacity, reduce gene mutation frequency, suppress the dissemination of resistance genes, and consequently enhance antibacterial efficiency.

*Staphylococcus aureus* is a bacterium that commonly exists on the skin and in the nasal passages of humans and animals. It is a versatile pathogen known to cause a wide range of infections, from mild skin infections like boils and abscesses to severe invasive infections, such as bloodstream infections, pneumonia, and surgical site infections. The excessive use of antibiotics in the clinical treatment of *S. aureus* infections has led to the prevalence of antibiotic‐resistant strains, such as methicillin‐resistant *S. aureus* (MRSA) mutants and SCVs^9^. Growing evidence suggests that the SOS response system significantly contributes to *S. aureus*'s resistance to host immune defenses and facilitates the emergence of drug‐resistant strains. However, current in‐depth research on proteins involved in bacterial SOS response systems primarily focuses on model species, such as *Escherichia coli* and *Bacillus subtilis*. The regulatory and effector proteins in the SOS response system within *S. aureus*, along with their regulatory networks and corresponding protein structures, may differ from those in these model bacteria. Therefore, determining drug targets for the SOS response system in *S. aureus* solely based on protein models from other bacterial species may not be sufficient.

In this review, we aim to provide an overview of the activation, process, and consequences of the SOS response in *S. aureus*. We have focused on summarizing the bioinformatics, structural information (utilizing experimental data or structures predicted using AlphaFold2 software), functions, molecular mechanisms, and potential inhibitors of *S. aureus* SOS response‐related proteins. Additionally, we have compared these proteins with those involved in the SOS response in *E. coli*. We hope this summary can provide insights into the clinical prevention and reduction of antibiotic resistance in *S. aureus*.

## ACTIVATION OF THE SOS RESPONSE IN *S. AUREUS*

**Figure 1:**
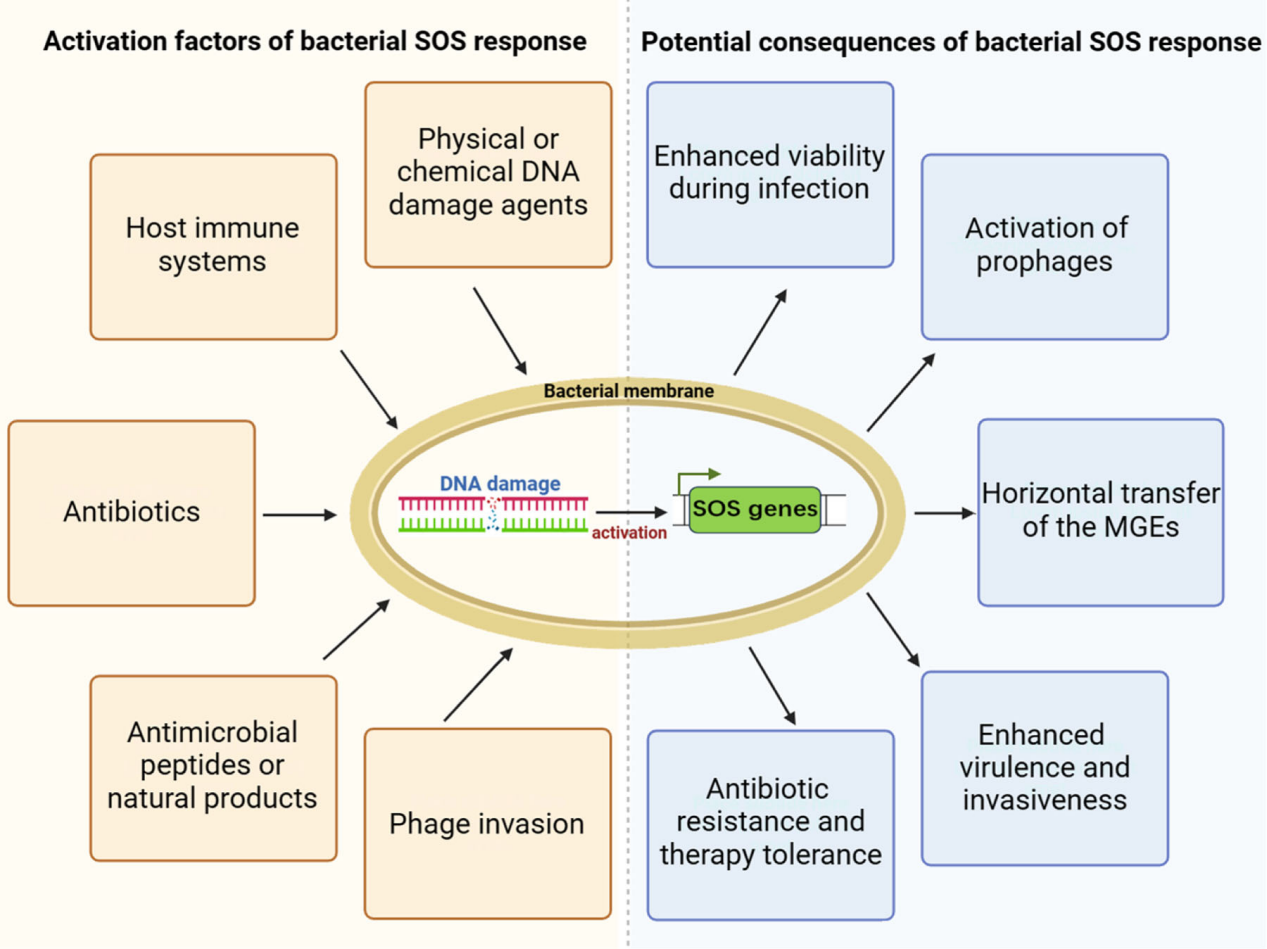
Activation factors and potential consequences of the bacterial SOS response. The major activation factors of the bacterial SOS response are summarized and highlighted with orange frames, while major potential consequences are highlighted with blue frames. MGEs, mobile genetic elements.

The bacterial SOS response system can be activated by a wide range of factors, including physical or chemical DNA‐damaging agents, host immune reactions, specific antibiotics, antimicrobial peptides, natural products, and phage infections. Despite their diversity, these factors share a common trait: their capacity to cause bacterial DNA damage, either directly or indirectly, consequently initiating the SOS response. In this context, we delve into the specific factors that potentially initiate the SOS response during the growth and infection phases of *S. aureus* (Figure 1).

### Physical or chemical DNA damage agents

The primary factors known to activate the bacterial SOS response system include exposure to physical DNA damage agents, such as ultraviolet (UV), X‐ray irradiation, gamma‐irradiation, and chemical DNA‐damaging agents like hydrogen peroxide (H_2_O_2_), methyl methanesulfonate (MMS), mitomycin C (MMC), paraquat, and others. In the case of *S. aureus*, experimental evidence has confirmed that the SOS response can be triggered by agents, such as H_2_O_2_, MMC, and paraquat, leading to DNA DSBs or the generation of detrimental ROS that inflict damage upon bacterial DNA^10^, ^11^, ^12^.

### Host immune systems

A crucial defense mechanism against *S. aureus* infection involves the respiratory burst of neutrophils. This burst generates ROS and causes various types of DNA damage, including DSBs^1^, ^13^, ^14^. Reports indicate that the processing of DNA DSBs activates the SOS response within 30 min of bacterial exposure to neutrophils^1^.

Phagocytosis of *S. aureus* by macrophages also triggers the SOS response^15^. The exact basis for this observation is still unknown. However, it is noteworthy that during phagocytosis by macrophages, amino acid starvation induces the stringent response, which includes the expression of genes associated with oxidative stress resistance, indicating that nutrient limitation might induce DNA damage through the endogenous generation of ROS^16^.

### Antibiotics

There are proposals suggesting that exposure of bacteria to antibiotics may trigger the SOS response by directly damaging DNA or disrupting metabolism, leading to the generation of endogenous ROS^17^, ^18^.

Various antibiotics, including bactericidal agents, such as ciprofloxacin, co‐trimoxazole, and specific beta‐lactams, as well as daptomycin and nitrofurantoin, and bacteriostatic antibiotics like chloramphenicol and linezolid, have been observed to induce the staphylococcal SOS response^1^, ^19^, ^20^, ^21^, ^22^, ^23^. Notably, the combination antibiotic co‐trimoxazole and the quinolone ciprofloxacin are known to cause DNA DSBs, disrupt DNA replication, and consequently activate the SOS response^1^, ^20^, ^22^, ^24^. Beta‐lactam antibiotics such as oxacillin can stimulate the production of endogenous ROS, probably by increasing the activity of the tricarboxylic acid (TCA) cycle, causing DNA damage and triggering the SOS response in *S. aureus* ^21^. Although daptomycin, nitrofurantoin, chloramphenicol, and linezolid also cause DNA damage and trigger the SOS response in *S. aureus*, the exact mechanisms are yet to be characterized^19^.

### Antimicrobial peptides or natural products

There is evidence indicating that certain antimicrobial peptides (AMPs), such as the lysine‐peptoid hybrid LP5, the fallaxin analog FL9, and 8 h‐CHH, can induce the SOS response in *S. aureus* by inhibiting DNA replication or causing iron dysregulation and membrane permeabilization^25^, ^26^, ^27^.

Colibactin is a chemically unstable small‐molecule genotoxin produced by several different bacteria^28^. Reports have suggested that colibactin possesses DNA‐alkylating ability, inducing interstrand DNA cross‐links and activating the bacterial SOS response in *S. aureus* ^29^.

Resveratrol, a naturally occurring phytoalexin, has been extensively studied for its potential health benefits, including antioxidant, anti‐inflammatory, anticancer, cardioprotective, neuroprotective, and antiaging properties^30^. Moreover, resveratrol has been confirmed to possess antimicrobial properties against bacteria, fungi, and viruses^31^. Treatment of *S. aureus* with subinhibitory concentrations of resveratrol increased the expression of SOS response genes, indicating its ability to activate the bacterial SOS response^32^.

Punicalagin, a natural metabolite from pomegranate, has been reported to disrupt iron homeostasis and induce the SOS response in *S. aureus*, possibly by inhibiting DNA biosynthesis.^33^

### Phage invasion

The bacterial SOS response can also be initiated by phage invasion. In response to phage attacks, certain bacteria deploy CRISPR‐Cas systems to elicit abortive infection phenotypes. This is achieved by activating indiscriminate nucleases that are unable to distinguish between self and invading DNA. These indiscriminate nucleases have the potential to disrupt bacterial DNA, triggering the SOS response. This phenomenon has been observed in types I‐E, I‐F, as well as types III and VI CRISPR‐Cas immunity systems^34^, ^35^. In the case of *S. aureus*, it has been reported that the infection by the staphylococcal lytic phage ϕNM1γ6 activates the nonspecific DNA degradation activity of Cas10 from the type III‐A CRISPR‐Cas system. This activation subsequently triggers the SOS response and accelerates the development of antibiotic resistance^36^.

## IMPACTS OF THE SOS RESPONSE ON *S. AUREUS*

The SOS response is a crucial process that not only influences bacterial survival within the host but also promotes genotypic variation through increased mutation rates and horizontal gene transfer (Figure 1). Ultimately, this leads to the emergence of strains capable of resisting host defenses and antibiotics. Below, we will describe in detail the potential impacts of the SOS response on *S. aureus*.

### Enhanced viability during infection

As discussed above, the adverse conditions encountered during infection, particularly the ROS produced by the host's immune response and antibiotics, can cause DNA damage and compromise the genomic stability of bacteria. Unrepaired damage may result in failed infection and cell death. The activation of the SOS response leads to the upregulation of numerous DNA repair proteins, a discussion of which will follow, facilitating the rapid repair of damaged DNA. This DNA repair process was found to be crucial for the survival of *S. aureus* in an ex vivo whole human blood model, as well as in both systemic and superficial murine infections^1^. Further evidence supporting the pivotal role of DNA repair in staphylococcal viability during infection comes from transposon sequencing (Tn‐seq) experiments. These experiments revealed that mutants lacking various DNA repair proteins, such as RexA, RexB, RecF, RecN, RecO, or RecF, were unable to survive in murine skin, soft tissue, or murine lung infection models^37^, ^38^.

Moreover, the induction of the SOS response leads to an increased mutation rate, primarily due to the expression of error‐prone DNA polymerase^12^, ^39^. This elevated mutation rate accelerates the emergence of staphylococcal SCVs^40^. SCVs arise from mutations in genes governing the electron transport chain, leading to reduced metabolic activity and the formation of small colonies due to slow growth^41^. Notably, SCVs were observed to exhibit resilience to neutrophil‐mediated killing^12^, ^39^.

### Activation of prophages

The genomes of numerous staphylococcal strains harbor prophages, which can become functional bacteriophages upon activation^42^. While activation can occur spontaneously, it often takes place during SOS induction, leading to the phage entering the lytic cycle^43^, ^44^. The DNA damage agent H_2_O_2_, certain antibiotics, and bacterial natural product colibactin were reported to activate the lytic replication of prophages by inducing the SOS response in *S. aureus* ^29^, ^44^, ^45^. The CI repressor was identified as responsible for repressing genes involved in the lytic cycle of λ phages in *S. aureus*. During the SOS response, the CI repressor undergoes auto‐cleavage, resulting in the derepression of the lytic cycle^46^.

### Horizontal transfer of the mobile genetic elements (MGEs)

About 15%–20% of the *S. aureus* genome consists of MGEs, including prophage, plasmids, *S. aureus* pathogenicity islands (SaPIs), transposons, integrative conjugative elements (ICEs), integrons, and staphylococcal chromosome cassettes (SCCs). The horizontal transfer of MGEs primarily occurs through bacteriophage transduction or conjugation.^47^

During the lytic cycle, fragments from both chromosomal and plasmid DNA, rather than phage DNA, can be packaged into the phage capsid. This results in the formation of a transducing particle that, when released from the donor cell, has the ability to transfer these DNA fragments to a recipient cell—a phenomenon commonly referred to as generalized transduction^48^. Ubeda et al. observed that exposure to ciprofloxacin prompted the dissemination of SaPIs, with efficiency significantly reduced upon the inactivation of the SOS response^49^. It was reported that the SOS response enhances the expression of encapsidation, facilitating the packaging of SaPI into phages^50^. SaPIbov1 can be transduced to several staphylococci other than *S. aureus* and even to different species, such as *Listeria monocytogenes*, which enhances the DNA transfer between *S. aureus* and other bacterial species^51^. In addition to SaPIs, it has been reported that gene transfer of plasmid DNA fragments and SCCmec elements of *S. aureus* can be mediated through SOS‐activated generalized transduction^52^, ^53^.

There is currently no evidence to support that conjugation‐mediated gene transfer can be directly controlled by the SOS response. However, it has been reported that the SOS response can lead to the formation of biofilm, which could enhance the efficiency of conjugation^54^.

### Enhanced virulence and invasiveness

Numerous lines of evidence have confirmed that the SOS‐mediated horizontal transfer of MGE genes contributes to the dissemination of SaPIs‐encoded virulence factors^43^, ^45^, ^49^.

Moreover, the SOS response plays a significant role in regulating various virulence factors produced by *S. aureus*. The SOS response activates the overexpression of staphylokinase on prophage φ13, a phage‐encoded virulence factor that not only inhibits the antibacterial activity of AMPs but also converts plasminogen to plasmin, further enhancing the virulence of *S. aureus* ^45^.

Additionally, the SOS response leads to an upsurge in the production of fibronectin‐binding protein B (encoded by the *fnbB* gene), consequently bolstering biofilm formation and enhancing its capacity to bind to host surfaces^55^.

Furthermore, during intracellular survival, the emergence of staphylococcal SCVs, stimulated by SOS response, contributes to biofilm development and leads to chronic and recurrent infections, posing a challenge for their treatment^56^, ^57^, ^58^.

### Antibiotic resistance and therapy tolerance

The induction of the SOS response, as anticipated from its impact on mutation rates, leads to higher instances of spontaneous resistance to rifampin, co‐trimoxazole, ciprofloxacin, and norfloxacin^20^, ^39^, ^59^, ^60^. Many MRSA strains exhibit heteroresistance to beta‐lactams, where only a small subpopulation of cells is phenotypically resistant to this class of antibiotics. However, beta‐lactams stimulate the induction of the SOS response through endogenous ROS production, leading to a transition from heteroresistance to homogeneous resistance^57^, ^61^, ^62^. The involvement of SOS‐induced mutations in this process has been confirmed by using ciprofloxacin to trigger SOS response, resulting in the emergence of homogeneous beta‐lactam resistance in *S. aureus* ^63^.

The SOS response has also been shown to disseminate antibiotic resistance by facilitating the horizontal transfer of resistance determinants on MGEs^6^, ^42^. In *S. aureus*, it has been reported that methicillin‐resistant genes on plasmid DNA and SCCmec elements can be transferred through SOS response‐activated generalized transduction, thereby enhancing the formation of methicillin‐resistant strains^52^, ^53^. In addition, *S. aureus* biofilms promote the horizontal transfer of antibiotic resistance by enhancing the efficiency of plasmid conjugation^54^.

Beyond antibiotic resistance, the SOS response might also play a role in bacteria's therapy tolerance. The SOS response induces the expression of the SosA protein, which inhibits cell growth and potentially contributes to the formation of persisters—nongrowing, antibiotic‐tolerant bacteria^15^, ^64^. In addition to leading to chronic and recurrent infections as described above, staphylococcal SCVs also exhibit a heightened level of antibiotic tolerance^56^, ^57^, ^58^. Moreover, the activation of the SOS response has been associated with the development of tolerance to photodynamic therapy in *S. aureus* ^65^.

## PROTEINS PARTICIPATING IN THE *S. AUREUS* SOS RESPONSE AND THEIR RELATED INHIBITORS

**Figure 2:**
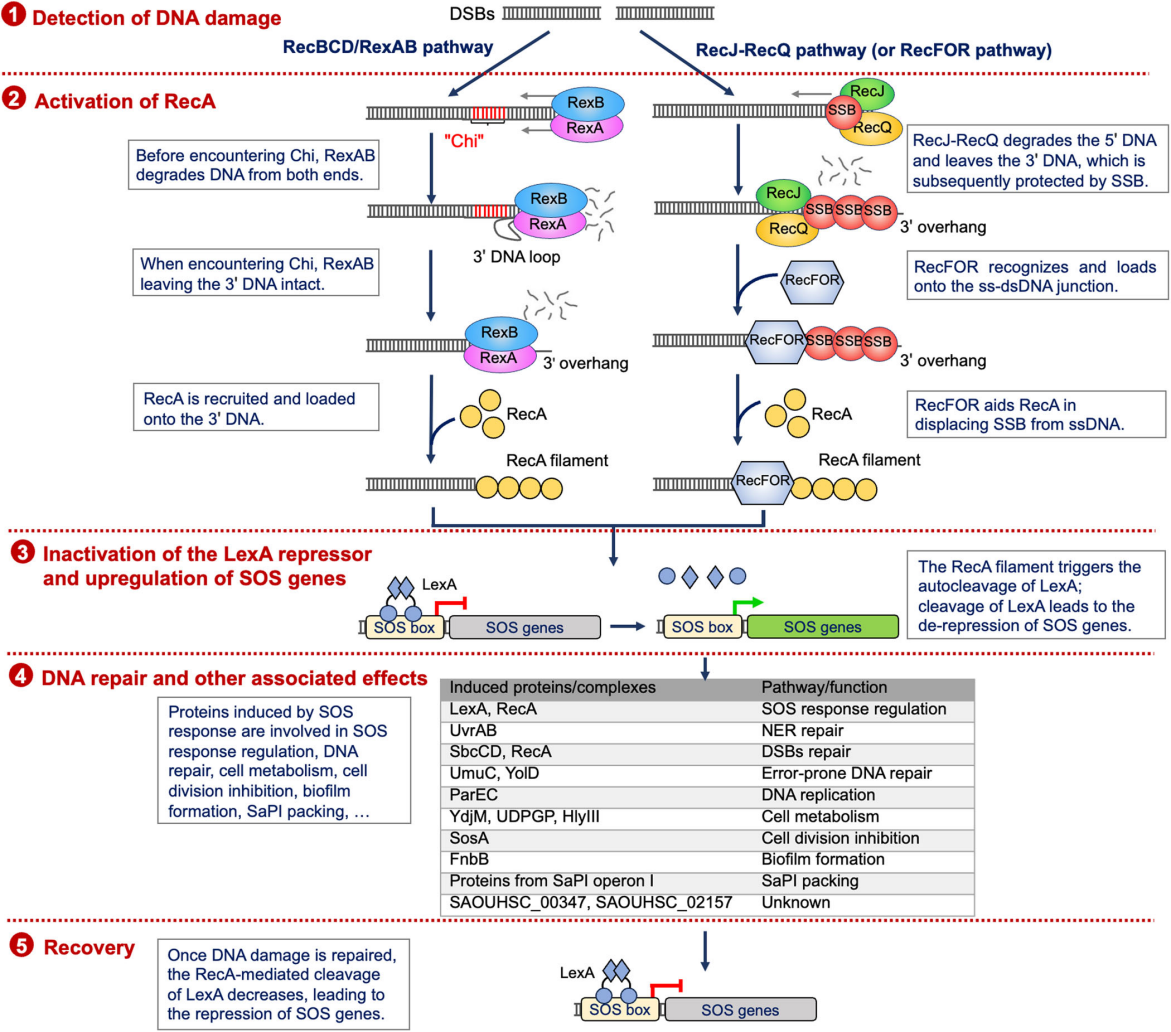
The SOS response pathway in *Staphylococcus aureus*. In the absence of DNA damage, the LexA repressor binds to the SOS box as dimers, suppressing the expression of downstream SOS genes. Upon the occurrence of DNA lesions, ssDNA arises from double‐strand breaks via pathways, such as RecBCD/RexAB or RecJ‐RecQ. This leads to the formation of RecA filaments, which trigger the autocatalytic cleavage of LexA. The truncated LexA loses its ability to bind to the SOS box, thereby enabling the expression of SOS genes. Derepressed SOS genes are involved in SOS response regulation, DNA repair, cell metabolism, inhibition of cell division, biofilm formation, and SaPI packing. Once DNA damage is repaired, the RecA‐mediated cleavage of LexA decreases, leading to the repression of SOS genes.

The complete process of the SOS response involves the detection of DNA damage, activation of RecA, inactivation of the LexA repressor, and upregulation of SOS genes, DNA repair, and other associated effects, followed by recovery (Figure 2).

Under normal conditions without DNA damage, LexA exists as a dimer and inhibits gene expression within the SOS regulon by binding to specific promoter regions known as SOS boxes (Figure 2). DNA damage induces the formation of single‐stranded DNA (ssDNA), either during the replication of damaged DNA templates or through processing by broken DNA end resection enzymes (RecBCD/AddAB family enzymes or RecJ‐RecQ complex). RecA interacts with the ssDNA, forming a nucleoprotein filament that activates LexA for self‐cleavage. The truncated LexA then dissociates from the SOS boxes, leading to the derepression of SOS genes^3^, ^4^ (Figure 2).

The proteins encoded by SOS genes participate in numerous cellular processes in *S. aureus*, including SOS response regulation, DNA repair, cell metabolism, cell division inhibition, biofilm formation, and SaPI packing.

In this context, we summarize the functions, structures (or predicted structures), and potential inhibitors for the proteins involved in each process of the SOS response within *S. aureus*, encompassing both regulatory and effector proteins.

### Regulation proteins of the SOS response in *S. aureus*

#### LexA repressor

The identification of LexA as a repressor within the SOS regulatory network in *E. coli* dates back to 1982^66^. The consensus DNA target recognized by *E. coli* LexA (EcoLexA), known as the SOS box, is CTGT‐N8‐ACAG, a sequence conserved in numerous Gram‐negative bacteria^67^, ^68^. In contrast, Gram‐positive bacteria typically display a consensus sequence of GAAC‐N4‐GTTC^67^. The distinctive consensus sequence for *S. aureus* LexA (SaoLexA) has been identified as CGAACAAATGTTCG, with underlined segments denoting high conservation^22^.

**Figure 3:**
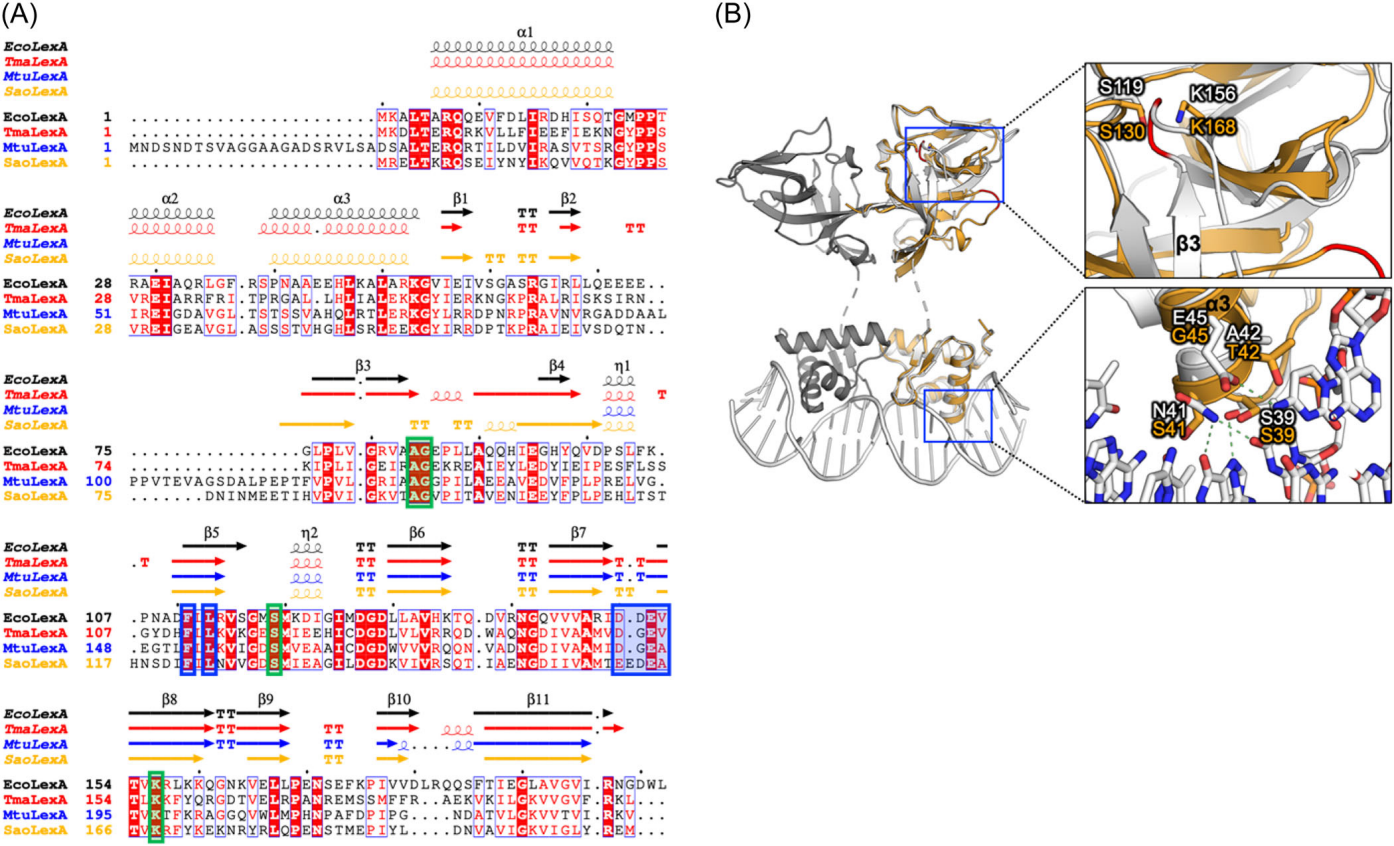
Sequence and structural analysis of *S. aureus* LexA. (A) Sequence alignments of the amino acids of structured LexAs and AlphaFold2‐predicted LexA from *S. aureus* NCTC8325. EcoLexA, *Escherichia coli* LexA (KEGG gene ID: b4043; PDB ID: 3JSO); TmaLexA, *Thermotoga maritima* MSB8 LexA (KEGG gene ID: TM1082; PDB ID: 3K2Z); MtuLexA, *Mycobacterium tuberculosis* H37Rv LexA (KEGG gene ID: Rv2720; PDB ID: 6A2Q); and SaoLexA, *S. aureus* NCTC8325 LexA (KEGG gene ID: SAOUHSC_01333; structure predicted by AlphaFold2). Secondary structural elements were depicted based on previously published structures or predictions in this work, shown at the top of the sequences. The cleavage site and critical residues for catalysis are highlighted in green boxes, while the residues crucial for LexA–RecA interaction are highlighted in blue boxes. (B) Superimposition of the structures of EcoLexA (depicted in white and gray) and the predicted SaoLexA (in orange) from AlphaFold2. The structures are shown as cartoons, with the (predicted) key residues for catalysis and DNA binding presented as sticks. The (predicted) cleavage sites are highlighted in red.

The structures lacking DNA binding of EcoLexA (PDB ID: 3JSO), *Thermotoga maritima* MSB8 LexA (PDB ID: 3K2Z), and *Mycobacterium tuberculosis* LexA (only C‐terminal domain (CTD), PDB ID: 6A2Q) have been reported^69^, ^70^. The interactions between EcoLexA and the corresponding SOS box have been elucidated through the analysis of crystal structures of EcoLexA‐DNA complexes^71^. EcoLexA binds the SOS box in a dimeric form. Each subunit comprises an N‐terminal domain (NTD, 1–69 aa) and a CTD (75–198 aa), connected through a hinge. The NTD of EcoLexA displays a helix‐turn‐helix (HTH) architecture consisting of three α‐helices and two β‐sheets. The CTD is entirely composed of β‐sheets. The cleavage site (A84‐G85 in EcoLexA) is situated in a flexible loop at the N‐terminus of the CTD and is highly conserved (Figure 3). The catalytic residues of the protease active site of EcoLexA, S119, and K156, which are extremely conserved among LexAs, are positioned in the core of the CTD (Figure 3). It was hypothesized that the RecA‐ssDNA filament would stabilize the LexA cleavage site, positioning an internal scissile peptide bond adjacent to its protease active site, and consequently stimulate the auto‐cleavage of LexA^72^. In each EcoLexA domain, the HTH motif engages with DNA in a canonical manner, where the recognition helix is positioned within the major groove while the wing interacts with the nearby minor groove (Figure 3B)^71^. The N‐terminal part of the recognition helix, α3, extends into the major groove and makes direct and/or water‐mediated interactions with all four base pairs. Protein–DNA interactions are primarily mediated by S39, N41, A42, and E45 on EcoLexA, and recognition of the conserved CTGT motif in the SOS box appears to depend heavily on direct readout (Figure 3B). Mutation of these residues altered specificity and sequence preference^71^, ^73^.

The geometry of each domain, catalytic residues, and cleavage site in the AlphaFold2‐predicted SaLexA structure is identical to that of EcoLexA. However, none of these DNA binding residues are conserved, which might explain why SaoLexA prefers to bind a different consensus sequence compared to EcoLexA. The cleavage site region of EcoLexA can enter the binding pocket and form a long, twisted hairpin that lies in the cleft and is presented to the catalytic S119 and K156. However, the cleavage site region of the predicted monomeric SaoLexA is located far away from the catalytic center. The intricate details regarding SaLexA dimerization and its interaction with the SOS box remain to be fully revealed.

The significance of autocatalytic LexA cleavage in initiating the SOS response in *S. aureus* was demonstrated by generating a strain bearing a point mutation on LexA, where a catalytically essential serine residue was replaced with alanine (S130A)^22^. When exposed to ciprofloxacin, this mutant strain exhibited a deficiency in initiating the SOS response, rendering it more susceptible to DNA damage induced by UV light or MMS^22^.

The self‐cleavage of LexA results in the separation of its NTD from the CTD, causing a 10–1000‐fold reduction in the DNA‐binding affinity of the NTD. Additionally, self‐cleavage triggers further degradation of LexA's NTD by ClpXp and/or ClpCp proteases, a process crucial for complete derepression^74^. Studies indicate that certain SOS genes in *S. aureus*, such as *sosA*, are derepressed only when the NTD undergoes additional digestion by Clp proteases^74^, ^75^. This process resembles the mechanism observed in *E. coli*, where ClpXp‐mediated degradation of LexA's NTD is essential for full induction of the SOS response and survival following DNA damage caused by UV exposure^68^, ^76^.

To date, only a few potential inhibitors of LexA have been identified. It was discovered that 5‐amino‐1‐(carbamoylmethyl)−1*H*‐1,2,3‐triazole‐4‐carboxamide can specifically inhibit the self‐cleavage activity of LexA in both *E. coli* and *Pseudomonas aeruginosa* ^77^, ^78^. In another study, small boron‐containing compounds, specifically phenylboronic derivatives, were shown to inhibit LexA self‐cleavage by forming an acyl‐enzyme intermediate with the catalytic Ser in *E. coli* ^79^. These inhibitors may also exhibit efficacy against *S. aureus* as they target the conserved catalytic center of LexA, shared among various bacterial species. Moreover, a triazole‐containing tricyclic inhibitor, mimicking the cleavage site of LexA, was reported to function by occluding the cleavable residues from the protease active site^72^.

It has been reported that the gp7 protein from *Bacillus* phage GIL01 reduced the SOS response by interacting with LexA, increasing the affinity of LexA‐SOS box, and reducing the autocleavage efficiency of LexA. Although from unrelated bacteria, gp7 exhibited robust interaction with SaoLexA and might show similar modulation of SOS response in *S. aureus* ^80^. The llama‐derived nanobody also reduced the autocleavage efficiency of LexA by directly binding to it, even though it possesses a completely different structural geometry from gp7^81^.

#### SOS inducer RecA

RecA was identified as an inducer or sensor of the SOS regulatory network^66^. It has been reported that EcoLexA undergoes self‐cleavage at alkaline pH values around 9.0; however, at neutral pH, activated *E. coli* RecA (EcoRecA) filaments are required to stimulate the cleavage of EcoLexA^82^. Sensing is mediated by unspecific binding of RecA to ssDNA fragments, generated either by DNA‐damage‐induced replication interruption or by enzymatic processing of broken DNA ends^83^. The expression of RecA, in turn, is regulated by LexA, as the binding of SaoLexA to the *S. aureus* RecA (SaoRecA) promoter has also been demonstrated, consistent with *recA* regulation in other systems^3^, ^55^.

**Figure 4:**
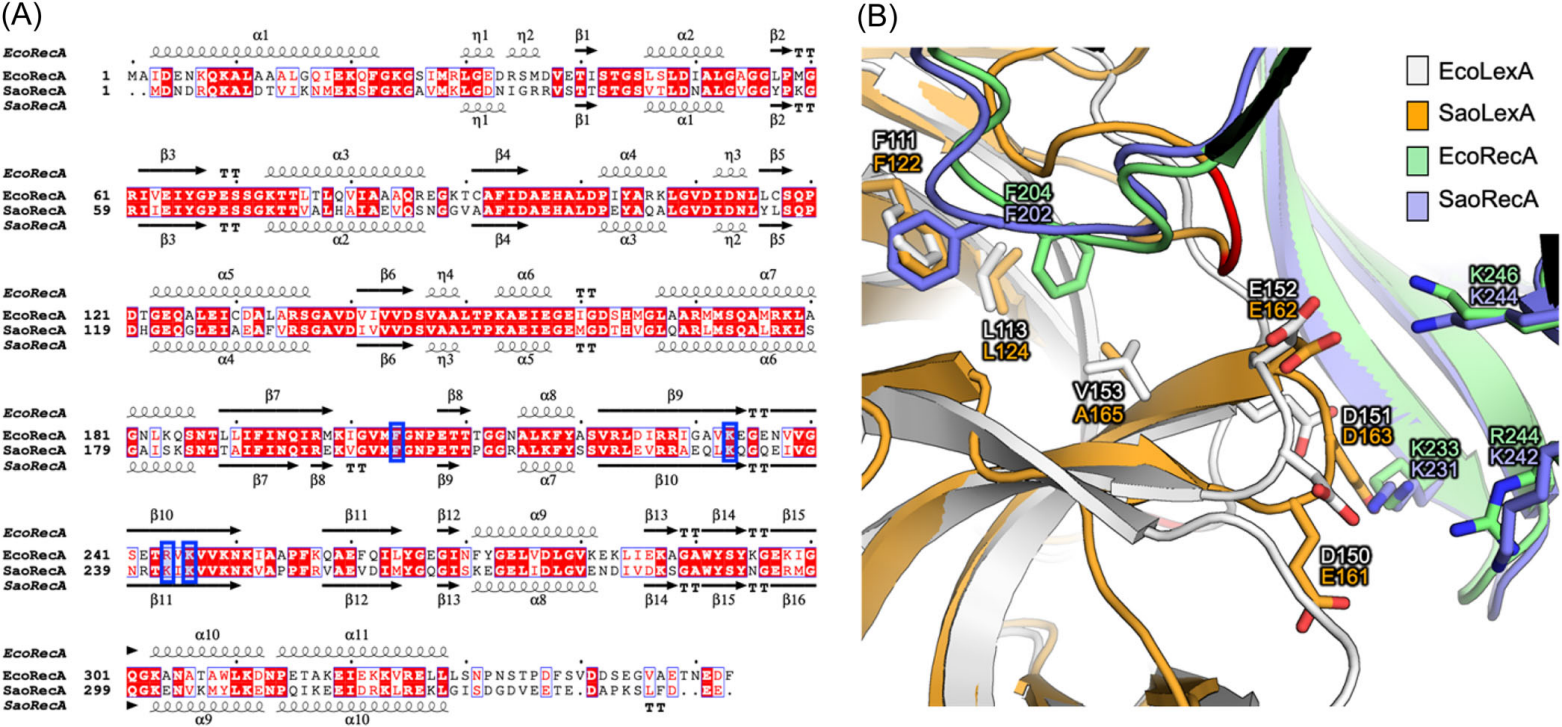
Sequence and structural analysis of *S. aureus* RecA. (A) Sequence alignments of EcoRecA and SaoRecA. EcoRecA, *E. coli* RecA (KEGG gene ID: b2699; PDB ID: 8GMS); SaoRecA, *S. aureus* NCTC8325 RecA (KEGG gene ID: SAOUHSC_01333; structure predicted by AlphaFold2). Secondary structural elements are depicted according to the structures published previously or predicted by AlphaFold2 in this work and displayed at the top or bottom of the sequences. The key residues for LexA–RecA interaction are highlighted in blue boxes. (B) Superimposition of the structures of EcoRecA–LexA complex (green and white for EcoRecA and EcoLexA, respectively) and the AlphaFold2‐predicted structures of SaoRecA and SaoLexA (slate and orange, respectively). Structures are shown as cartoons. The (predicted) key residues for interactions are shown as sticks. The (predicted) cleavage sites on LexA are colored red.

RecA comprises canonical RecA‐type ATPase domains that contain conserved Walk A and Walk B motifs, along with a CTD^84^ (Figure 4A). The crystal structure of the EcoRecA‐ADP·AlF_4_‐ssDNA filament (PDB ID: 3CMU) indicates that RecA binds to ssDNA in an ATP‐dependent manner, forming a helical nucleoprotein filament with approximately 6.2 RecA proteins per turn and roughly three nucleotides per RecA protein^85^. SaoRecA and EcoRecA share a 59% sequence identity. While experimental structural information on SaoRecA is not available, the AlphaFold2‐predicted SaoRecA structure shares a similar architecture with EcoRecA (Figure 4B).

The structure of EcoLexA in complex with EcoRecA filament (PDB ID: 8GMS) indicates that the catalytic CTD of LexA directly binds to RecA^86^. This interaction is primarily characterized by hydrophobic and electrostatic interactions. In terms of hydrophobic interaction, the residues F204 of EcoRecA inserts into a hydrophobic pocket formed by EcoLexA residues F111, L113, and V153. In terms of electrostatic interactions, a cluster of negatively charged residues on EcoLexA (D150, D151, and E152) interacts with a patch of positively charged residues (K233, R244, and K246) in the groove of EcoRecA filaments^86^. These key residues for protein‐protein interactions are conserved in SaoLexA and SaoRecA (Figure 4).

Deletion of *recA* in both *E. coli* and *S. aureus* led to a substantial reduction in antibiotic‐induced resistance^87^. Hence, targeting RecA for suppression or attenuation of the SOS response system has been proposed as a potential therapeutic strategy to suppress the development of antibiotic multiresistance and resistance‐conferring mutagenesis^88^.

To date, numerous RecA inhibitors have been reported, mainly based on the mechanism of inhibiting the ssDNA‐dependent ATPase activity of RecA. In vitro, the ATPase activity of RecA can be inhibited by ADP and various nonhydrolyzable ATP derivatives, such as N^6^‐(1‐naphthyl)‐ADP^89^. Three polysulfated naphthyl compounds, congo red, suramin, and bis‐ANS, which are compounds related to the nonnucleotide inhibitors of purine nucleotide receptors, have been reported to inhibit EcoRecA's ATPase activity^90^. Suramin, in particular, has been found to abolish ciprofloxacin‐induced *recA* gene expression and the SOS response, thereby enhancing the bactericidal action of ciprofloxacin^91^. Moreover, 2‐amino‐4,6‐diarylpyridine, as well as other 35 compounds were screened as RecA ATPase inhibitors in *E. coli* ^92^, ^93^.

Uncompetitive inhibitors targeting RecA's ATP binding site have also been identified, including some Lichen secondary metabolites, such as divaricatic, perlatolic, alpha‐collatolic, lobaric, lichesterinic, protolichesterinic, epiphorellic acids, sphaerophorin, and tumidulin, along with natural phenolic compound gallic acid and p‐Coumaric acid, and inhibitors based on phthalocyanine tetrasulfonic acid^94^, ^95^, ^96^, ^97^. These anionic and aromatic molecules are thought to engage in cation‐anion interactions with RecA, competitively obstructing its DNA binding. Consequently, they effectively impede RecA‐mediated processes, such as DNA binding, DNA strand exchange, RecA filament formation, LexA autoproteolysis, and SOS induction. Notably, gallic acid has been observed to hinder ciprofloxacin‐induced RecA expression, thereby playing an inhibitory role in the SOS survival mechanism in *S. aureus* ^96^. Inhibitors based on phthalocyanine tetrasulfonic acid have demonstrated efficacy in preventing the acquisition of ciprofloxacin resistance in a murine thigh infection model (mice thighs infected with pathogenic *E. coli* ATCC25922), highlighting the potential of targeting the SOS response^97^.

Besides, zinc salts have been confirmed to interfere with RecA actions, protecting LexA from RecA‐mediated autocleavage, thereby inhibiting the SOS response and the hypermutator phenomenon in *E. coli* as well as in *Klebsiella pneumoniae* ^98^.

However, it is important to note that RecA homologs exist in humans (Rad51 and Dmc1)^99^, raising concerns about potential host toxicity with RecA inhibitors. To circumvent potential host toxicity, certain peptides specifically targeting RecA have been proposed as RecA inhibitors. For example, a 29‐mer peptide designed based on the NTD of EcoRecA, involved in intermonomer contact, inhibits EcoRecA filament assembly^100^. A short peptide derived from *E. coli* RecX could both inhibit EcoRecA protein activities in vitro and block the bacterial SOS‐response in vivo^101^. Besides, the F plasmid PsiB protein inhibits the SOS response by suppressing all the activities of the RecA protein through protein–protein interaction^102^.

#### RexAB

As mentioned above, RecA filaments assist in searching for a homologous DNA sequence for strand invasion and repair, as well as triggering LexA autocleavage. The formation of RecA filaments requires the generation of 3′‐end free ssDNA through DNA end resection, a process mediated by a specific DNA helicase–nuclease complex. Bacteria primarily utilize two major complexes for DNA end resection: RecBCD/RexAB and RecJ‐RecQ. The NurA–HerA helicase–nuclease complex, present in only a limited number of bacteria, has also been identified to contribute to DNA end resection^103^, ^104^. *S. aureus* possesses RecBCD/RexAB and RecJ–RecQ but lacks the NurA–HerA complex.

In the case of RecBCD/RexAB (also known as AddAB in *B. subtilis* and *Helicobocton pyloni*), they initially recognize and bind to the broken DNA end. Subsequently, they initiate the unwinding of the DNA duplex using their helicase activity, resulting in the generation of an ssDNA region. Concurrently, the nuclease activity of RecBCD or RexAB degrades the unwound DNA strand, producing a 3′‐overhang in a Chi sequence‐regulated manner. The resulting 3′‐end free ssDNA tail serves as a substrate for direct RecA loading.

**Figure 5:**
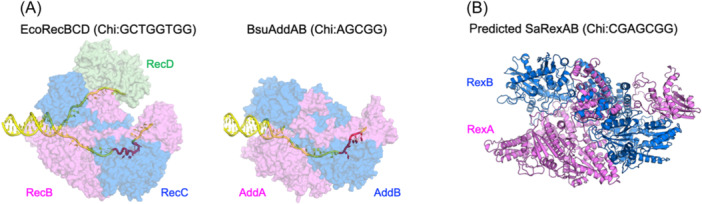
Structural analysis of *E. coli* RecBCD, *Bacillus subtilis* AddAB, and *S. aureus* RexAB. (A) Comparison of EcoRecBCD and BsuAddAB Chi‐bound complexes. Transparent surfaces of EcoRecBCD (left; PDB ID:6SJB) and BsuAddAB (right; PDB ID:4CEI) were colored by subunit, with the DNA backbones overlaid as a cartoon in yellow with their respective Chi sequences depicted in red. (B) The AlphaFold2‐predicted SaRexAB complex structure. The RexA and RexB subunits are depicted in cartoon form, colored violet and marine, respectively.

RecBCD is predominantly found in Gram‐negative bacteria, constituting a three‐subunit complex with a nuclease domain and two helicase domains, while RexAB/AddAB is present in Gram‐positive bacteria, forming a two‐subunit complex with two nuclease domains and one helicase domain^105^. The complex structure of *E. coli* RecBCD (EcoRecBCD) with the Chi sequence reveals a highly coiled binding of the Chi sequence within the RecC subunit, mainly mediated by base‐amino acid interactions, uncovering the mechanism by which RecBCD specifically recognizes the Chi sequence (Figure 5A). Chi sequence binding induces conformational changes in the RecB nuclease domain and part of RecC, implying that Chi influences RecBCD activity by altering its structure^106^. On the other hand, the resolved structure of the *B. subtilis* AddAB (BsuAddAB) in complex with the Chi sequence shows that the Chi sequence binding within the AddB subunit is relatively flat (Figure 5A), suggesting a somewhat different Chi recognition and regulatory mechanism^107^. The predicted structure of *S. aureus* RexAB (SaRexAB) resembles BsuAddAB and might share a similar Chi‐dependent regulatory mechanism (Figure 5B).

SaRexAB has been proven to be crucial for bacterial infection and survival when exposed to multiple classes of antibiotics^1^, ^19^. A mutant defective for *rexB* in *S. aureus* showed increased antibiotic susceptibility and was unable to induce the SOS response when exposed to antibiotics^1^, ^19^.

Various inhibitors targeting RecBCD/AddAB have been explored. RecBCD inhibitors include compounds like adozelesin, ecteinascidin 743, hedamycin, cisplatin, and psoralen^108^, ^109^, ^110^. Amundsen et al. identified 21 potential inhibitors for EcoRecBCD and *H. pylori* AddAB. Among these, CID 1517823 displayed the highest activity against EcoRecBCD, while CID 697851 proved to be the most effective against *H. pylori* AddAB^111^. However, these inhibitors face challenges, such as limited in vivo stability, low oral bioavailability, and suboptimal mechanisms of action involving DNA alkylation, rendering them nonselective and highly cytotoxic^108^, ^109^, ^110^. Additionally, certain bacteriophage proteins, like the Gam protein from bacteriophage lambda^112^, the gp5.9 protein from bacteriophage T7, and the Abc2 protein from *Salmonella* phage P22^113^, are known to inhibit the activity of RecBCD.

Homologs of RecBCD/RexAB are prevalent in over 90% of sequenced bacteria^114^. This suggests that targeting RecBCD/RexAB could potentially serve as a broad‐spectrum therapeutic approach. Furthermore, as RecBCD/RexAB homologs are absent in eukaryotes, the risk of RecBCD/RexAB inhibitors affecting host protein is reduced.

Of note, there are significant differences in the Chi sequences of this system across different species. For instance, EcoRecBCD can specifically recognize the sequence “GCTGGTGG”^105^. BsuAddAB can specifically recognize the sequence “AGCGG”^115^, while SaRexAB can specifically recognize “GAAGCGG”^116^. Therefore, designing inhibitors targeting specific Chi recognition and binding sites could provide insights into the development of narrow‐spectrum antibiotics.

#### RecJ–RecQ nuclease–helicase, SSB, and RecFOR mediator

Some bacteria naturally lack RecBCD and instead use RecJ–RecQ nuclease–helicase for generating 3′‐end free ssDNA. The single‐stranded binding protein, SSB, then coats the generated ssDNA. Subsequently, the RecFOR complex facilitates RecA loading onto the 3′‐end free ssDNA, probably by displacing SSB from the ssDNA^117^.

RecJ functions as a 5′−3′ directional ssDNA exonuclease. Assisted by the 3′−5′ helicase RecQ and SSB, it processes various forms of damaged DNA ends into elongated 3′ ssDNA ends^118^, ^119^. Although the 5′−3′ ssDNA exonuclease mechanism of RecJ has been elucidated through structural and biochemical methods previously^118^, ^120^, it remains unclear how RecJ efficiently collaborates with proteins like RecQ, SSB, and so forth, to recognize and degrade damaged DNA ends. Both RecJ and RecQ can bind to SSB, despite not having a direct interaction with each other. Moreover, SSB can enhance the exonuclease activity of RecJ and the helicase activity of RecQ by aiding DNA melting and binding in *E. coli* and *Deinococcus radiodurans* ^118^, ^119^. It is currently unknown whether there exists a cooperative binding among the three proteins to activate each other's activities. Additionally, how RecJ appropriately halts or slows down its DNA degradation activity at specific positions remains unknown. While it has not been reported whether RecJ possesses the ability to recognize specific Chi sequences like RecBCD or RexAB, previous studies on the activity of RecJ in *E. coli* and *D. radiodurans* have shown that RecJ exhibits different cutting efficiencies on DNA strands with different bases^118^, ^119^. Therefore, it is possible that the activity of RecJ is regulated by potential Chi sequences.

**Figure 6:**
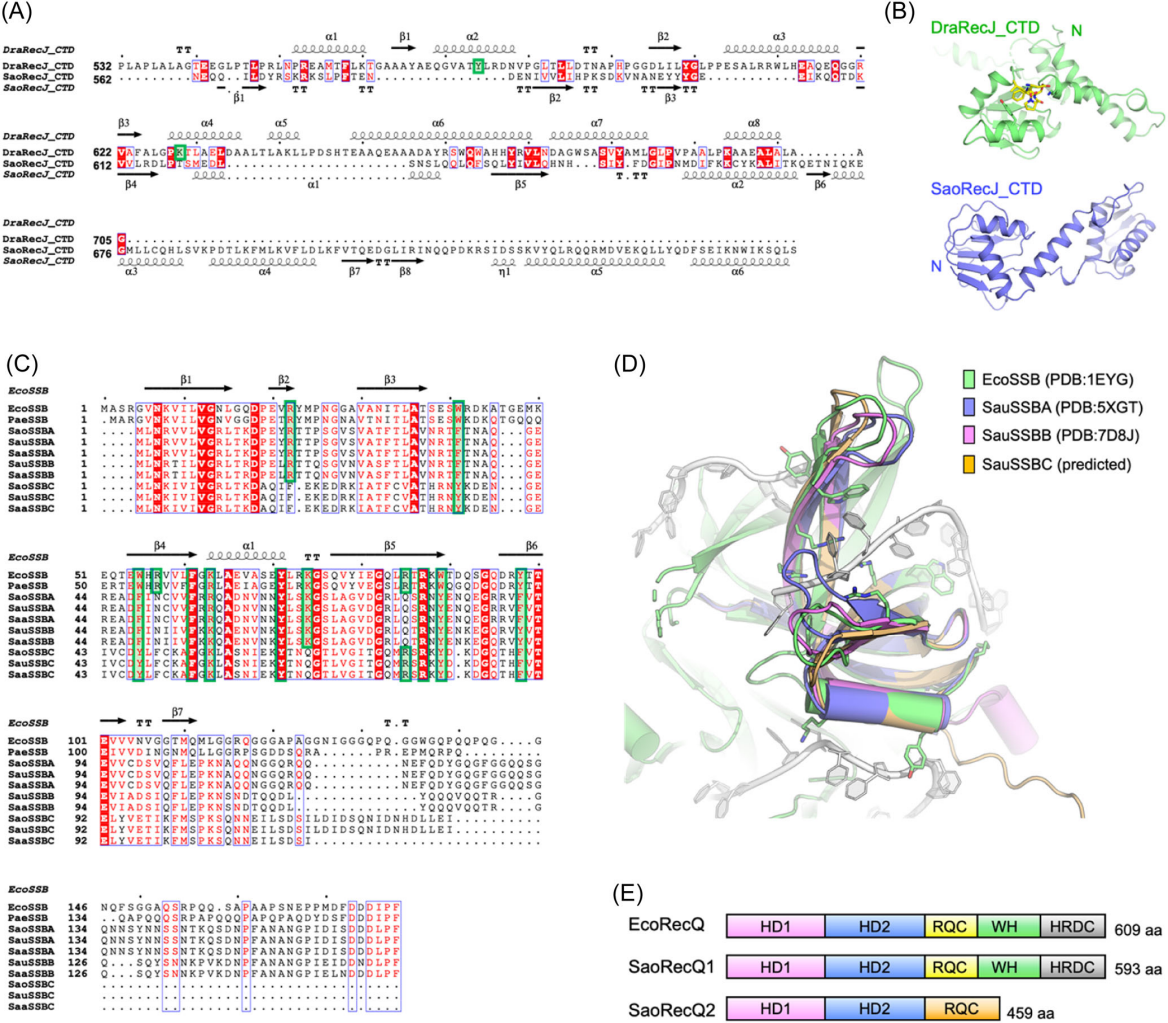
Sequence and structural analysis of *S. aureus* RecJ, SSB, and RecQ. (A) Sequence alignments of the *Deinococcus radiodurans* RecJ (DraRecJ; PDB ID: 5F56; KEGG gene ID: DR_1126) and the *S. aureus* NCTC8325 RecJ (SaoRecJ; KEGG gene ID: SAOUHSC_01744). Secondary structural elements were depicted according to the structures published previously or predicted by AlphaFold2 in this work, and displayed at the top or bottom of the sequences. (B) The structures of the CTD of DraRecJ (lime green; PDB ID: 5F56) and SaoRecJ (slate; predicted by AlphaFold2). The C‐terminal SSB peptide was colored yellow, and shown as sticks, as well as the key interaction residues on DrRecJ. (C) Sequence alignments of *E. coli* SSB (EcoSSB, PDB ID: 1EYG; KEGG gene ID: b4059), *Pseudomonas aeruginosa* SSB (PaeSSB; KEGG gene ID: PA4232), and SSBs from three different strains of *S. aureus*. Sao, *S. aureus* NCTC8325 (the KEGG gene ID for SaoSSBA and SaoSSBC are SAOUHSC_00349 and SAOUHSC_02334, respectively); Sau, *S. aureus* N315 (the KEGG gene ID for SauSSBA, SauSSBB, and SauSSBC are SA0353, SA1792, and SA1899, respectively); Saa, *S. aureus* USA300 (the KEGG gene ID for SaaSSBA, SaaSSBB, and SaaSSBC are SAUSA300_0367, SAUSA300_1958, and SAUSA300_2052, respectively). Secondary structural elements were depicted according to the published EcoSSB and displayed at the top of the sequences. The key DNA binding residues for catalysis are highlighted in green boxes. (D) Superimposing the structures of EcoSSB–ssDNA complex (green and white for EcoSSB and ssDNA, respectively; PDB ID: 1EYG), SauSSBA (slate; PDB ID: 5XGT), SauSSBB (violet; PDB ID: 7D8J), and SauSSBC (orange, predicted by AlphaFold2). Structures are shown in cartoons. The bases and key residues for DNA binding are shown as sticks. (E) Schematic of the domain arrangements of *E. coli* RecQ (EcoRecQ; KEGG gene ID: b3822), *S. aureus* NCTC8325 RecQ1 (KEGG gene ID: SAOUHSC_00730), and RecQ2 (KEGG gene ID: SAOUHSC_01502).

It has been reported that *S. aureus* lacking the RecJ protein shows increased sensitivity to resveratrol, a compound known to induce bacterial SOS response^32^. Similar to the *D. radiodurans* RecJ (DraRecJ), the *S. aureus* RecJ (SaoRecJ) harbors a nonconserved CTD, with significant differences observed in their respective sequences (Figure 6A). A binding pocket for the C‐terminal tail of SSB was identified on DraRecJ's CTD through structural and biochemical studies^118^. However, the AlphaFold2‐predicted SaoRecJ structure lacks such an SSB binding pocket (Figure 6B).

In *S. aureus*, there are typically three SSB proteins, referred to as SSBA, SSBB, and SSBC, respectively^121^, ^122^ (Table 1). SSBAs are the most conserved among different *S. aureus* subspecies and closely resemble *E. coli* SSB (EcoSSB). SSBB is absent in some *S. aureus* subspecies, such as the *S. aureus* NCTC8325 strain (Table 1). Sequence alignments and crystal structures of *S. aureus* N315 SSBA (SauSSBA) (PDB ID: 5XGT) and *S. aureus* N315 SSBB (SauSSBB) (PDB ID: 7D8J) indicate that, although considerably shorter in amino acid sequence than SauSSBA, SauSSBB shares a similar structure and moderate homology to SauSSBA, particularly in the classic oligonucleotide/oligosaccharide‐binding fold (OB fold), which is responsible for ssDNA binding^121^, ^122^, ^123^ (Figure 6C,D). SSBC is even shorter. The AlphaFold2‐predicted structure of *S. aureus* N315 SSBC (SauSSBC) indicates that it also comprises the classic OB fold, similar to SSBA and SSBB, at the N‐terminus (Figure 6D). The flexible C‐terminal tails of SSBCs are less conserved, typically less acidic, and 30–50 amino acids shorter than those of SSBAs and SSBBs (Figure 6C).

**Table 1:** Genes/proteins associated with the SOS response in *S. aureus* and potential inhibitors.

Gene/protein names and roles in *S. aureus*	KEGG gene ID			Protein structure in *S. aureus* (PDB ID)	Gene/protein names and roles in *E. coli*	Protein structures in *E. coli* (PDB ID)	Identified or potential inhibitors
	*S. aureus* NCTC8325	*S. aureus* N315 (MRSA/VSSA)	*S. aureus* USA300 (CA‐MRSA)				
**The SOS response repressor**							
*lexA*/LexA repressor, repressing the transcription of SOS genes by binding to the SOS boxes	SAOUHSC_01333	SA1174	SAUSA300_1237	NA	*lexA*/LexA repressor, represses the transcription of SOS genes by binding to the SOS boxes	LexA in complex with SOS box (3JSO, 3JSP)	5‐amino‐1‐(carbamoylmethyl)‐1H‐1,2,3‐triazole‐4‐carboxamide^77^, ^78^; small boron‐containing compounds (phenylboronic derivatives)^79^; triazole‐containing tricyclic inhibitor^72^; *Bacillus* phage GIL01 gp7^80^.
**The SOS response inducer/sensor**							
*recA*/RecA recombinase, regulating the autocleavage of LexA and activating the transcription of SOS genes, and required for recombinational repair	SAOUHSC_01262	SA1128	SAUSA300_1178	NA	*recA*/RecA recombinase, regulates the autocleavage of LexA and activates the transcription of SOS genes, and is required for recombinational repair	RecA in complex with ssDNA (3CMW); RecA in strand exchange reaction mode (7JY8)	ADP and different nonhydrolyzable derivatives of ATP, such as N^6^‐(1‐naphthyl)‐ADP^89^; polysulfated naphthyl compounds, such as congo red, suramin, and bis‐ANS^90^; 2‐amino‐4,6‐diarylpyridine, as well as another 35 compounds^92^, ^93^; Lichen secondary metabolites such as divaricatic, perlatolic, alpha‐collatolic, lobaric, lichesterinic, protolichesterinic, epiphorellic acids, p‐coumaric acid, sphaerophorin, and tumidulin, along with natural phenolic compound gallic acid and p‐coumaric acid, and inhibitors based on phthalocyanine tetrasulfonic acid^94^, ^95^, ^96^, ^97^; zinc salts^98^; a 29‐mer peptide designed based on the NTD of EcoRecA^100^; a short peptide derived from EcRecX^101^; F plasmid PsiB protein^102^.
**Genes/proteins involved in upstream regulation of SOS response**							
*rexA* a/RexA, a subunit of the DNA end resection complex RexAB	SAOUHSC_00905	SA0828	SAUSA300_0870	NA	*recB* a/RecB, a subunit of the broken DNA end resection complex RecBCD	RecBCD in complex with Chi‐contained DNA (6SJB, 6SJF)	Dozelesin, ecteinascidin 743, hedamycin, cisplatin, and psoralen^108^, ^109^, ^110^; 21 potent inhibitors found by Amundsen et al.^111^; bacteriophage lambda gam, T7 gp5.9 and P22 Abc2^112^, ^113^.
*rexB* a/RexB, a subunit of the broken DNA end resection complex RexAB	SAOUHSC_00904	SA0827	SAUSA300_0869	NA	*recC* a/RecC, a subunit of the broken DNA end resection complex RecBCD	RecBCD in complex with Chi‐contained DNA (6SJB, 6SJF)
NA	NA	NA	NA	NA	*recD* a/RecD, a subunit of the broken DNA end resection complex RecBCD	RecBCD in complex with Chi‐contained DNA (6SJB, 6SJF)
*recJ* a */*RecJ, probably 5′‐3′ exonuclease for broken DNA end resection, in coordination with RecQ	SAOUHSC_01744	SA1462	SAUSA300_1592	NA	*recJ* a */*RecJ, 5′–3′ exonuclease for broken DNA end resection, in coordination with RecQ	NA	NA
*recQ1* b/RecQ1, probably 3′‐5′ helicase for broken DNA end DNA resection, in coordination with RecJ; *recQ2* a */*RecQ2, unknown function	SAOUHSC_00730 SAOUHSC_01502	SA0676 SA1313	SAUSA300_0705 SAUSA300_1371	NA	*recQ* a/RecQ, 3′–5′ helicase for broken DNA end resection, in coordination with RecJ	RecQ catalytic core bound to ATP‐gamma‐S (1OYY)	NA
*ssbA* a */*SSBA, ssDNA binding protein	SAOUHSC_00349	SA0353	SAUSA300_0367	Apo structure (5XGT)	*ssb*/SSB, ssDNA binding protein that plays roles in DNA replication and repair	Apo structure (4MZ9)	Myricetin^124^; 9‐methyl‐2,3,7‐trihydroxy‐6‐fluorone (NSC5426)^123^; numerous inhibitors of SSB‐protein interactions^125^, ^126^.
*ssbB* a */*SSBB, ssDNA binding protein	NA	SA1792	SAUSA300_1958	Apo structure (7D8J)	NA	NA	
*ssbC* a */*SSBC, ssDNA binding protein	SAOUHSC_02334	SA1899	SAUSA300_2052	NA	NA	NA	
*recF* a */*RecF, a subunit of RecFOR, probably required for the formation of RecA‐ssDNA filament	SAOUHSC_00004	SA0004	SAUSA300_0004	NA	*recF* a */*RecF, a subunit of RecFOR, probably required for the formation of RecA‐ssDNA filament	NA	NA
*recO* a */*RecO, a subunit of RecFOR, probably required for the formation of RecA‐ssDNA filament	SAOUHSC_01667	SA1395	SAUSA300_1526	NA	*recO* a */*RecO, a subunit of RecFOR, probably required for the formation of RecA‐ssDNA filament	RecO in complex with SSB C‐terminus (3Q8D)	NA
*recR* a */*RecR, a subunit of RecFOR, probably required for the formation of RecA‐ssDNA filament	SAOUHSC_00445	SA0438	SAUSA300_0454	NA	*recR* a */*RecR, a subunit of RecFOR, probably required for the formation of RecA‐ssDNA filament	NA	NA
**Genes regulated by LexA**							
*uvrA*/UvrA, a subunit of ABC excinuclease	SAOUHSC_00780	SA0714	SAUSA300_0742	NA	*uvrA*/UvrA, a subunit of ABC excinuclease	Partial UvrA in complex with transcription‐repair‐coupling factor (4DFC)	2‐[5‐(methylamino)‐1,3,4‐thiadiazol‐2‐yl]‐3H‐benzo[f]chromen‐3‐one (ATBC)^127^.
*uvrB*/UvrB, a subunit of ABC excinuclease	SAOUHSC_00779^a^, SAOUHSC_00776	SA0713	SAUSA300_0741	NA	*uvrB*/UvrB, a subunit of ABC excinuclease	NA
*parC*/ParC, the subunit A of DNA topoisomerase IV	SAOUHSC_01352	SA1189	SAUSA300_1251	NA	*parC*/ParC, the subunit A of DNA topoisomerase IV	parC apo structure (1ZVU)	Fluoroquinolone antibiotics^128^; novel DNA intercalating bacterial topoisomerase inhibitors with alternative targeting sites as fluoroquinolone^129^, ^130^; kibdelomycin (KBD)^131^; Fic‐2 and Fic‐1^132^.
*parE*/ParE, the subunit B of DNA topoisomerase IV	SAOUHSC_01351	SA1188	SAUSA300_1250	ParE 43 kDa fragment in complex with KBD (4URL)	*parE*/ParE, the subunit B of DNA topoisomerase IV	ParE 43 kDa subunit complexed with ADPNP (1S16)	
*sbcC*/SbcC, the subunit C of SbcCD nuclease	SAOUHSC_01343	SA1181	SAUSA300_1243	NA	*sbcC* a/SbcC, the subunit C of SbcCD nuclease	SbcCD in complex with substrates (6S85)	NA
*sbcD*/SbcD, the subunit D of SbcCD nuclease	SAOUHSC_01341	SA1180	SAUSA300_1242	The nuclease and capping domain of SbcD (7DOG)	*sbcD* a/SbcD, the subunit D of SbcCD nuclease	SbcCD in complex with substrates (6S85)	NA
*umuC*/UmuC, probably the large subunit of the error‐prone DNA polymerase V	SAOUHSC_01363	SA1196	SAUSA300_1259	NA	*umuC*/UmuC, the large subunit of the error‐prone DNA polymerase V, translesion DNA synthesis	NA	NA
*yolD*/YolD, predicted to play a similar function as UmuD, is probably the small subunit of the error‐prone DNA polymerase V	SAOUHSC_02144	SA1738	SAUSA300_1903	NA	NA	NA	NA
NA	NA	NA	NA	NA	*umuD*/UmuD, the small subunit of the error‐prone DNA polymerase V, translesion DNA synthesis	UmuD in complex with RecA filament (8GMT)	NA
*sosA*/SosA, containing the SurA_N motif, a protein that inhibits cell division, possibly to allow time for DNA repair	SAOUHSC_01334	SA1175	NA	NA	NA	NA	NA
NA	NA	NA	NA	NA	*sulA*/SulA, encoding protein that inhibiting cell division, possibly to allow time for DNA repair	NA	NA
No specific gene name/encoding a hypothetical protein	SAOUHSC_00347	SAS009	SAUSA300_0365	NA	NA	NA	NA
No specific gene name/encoding hypothetical protein	SAOUHSC_02157	NA	NA	NA	NA	NA	NA
No specific gene name/containing a HlyIII motif, putative hemolysin III	SAOUHSC_02422	SA1973	SAUSA300_2129	NA	*yqfA* a/YqfA, hemolysin‐III family protein	NA	NA
No specific gene name/containing a UDPGP motif, putative uridylyltransferase	SAOUHSC_02423	SA1974	SAUSA300_2130	NA	NA	NA	NA
No specific gene name/containing a YdjM motif, conserved hypothetical protein	SAOUHSC_02424	SA1975	SAUSA300_2131	NA	*ydjM/*YdjM, inner membrane protein	NA	NA
fnbB/fibronectin‐binding protein B, related to biofilm formation.	SAOUHSC_02802	SA2290	SAUSA300_2440	NA	NA	NA	NA
Phage terminase small subunit	SAOUHSC_02050	NA	NA	NA	NA	NA	NA
Phage terminase large subunit	SAOUHSC_02049	NA	NA	NA	NA	NA	NA
Phage portal protein	SAOUHSC_02048	NA	NA	NA	NA	NA	NA

“NA” means “not appeared” or “not applicable.” ^a^These genes were not identified to be regulated by the LexA repressor^22^, ^133^. ^b^These genes contain putative SOS boxes, but additional confirmation is needed to determine whether they are regulated by the LexA repressor.

High‐throughput screening has been conducted to identify compounds inhibiting the binding of ssDNA to EcoSSB and SSBA^134^. Several compounds that inhibit SSBA also hinder the binding of SSBB to ssDNA in vitro^134^. The myricetin‐bound complex structure of *P. aeruginosa* PAO1 SSB (PaeSSB) revealed that myricetins occupy the grooves for ssDNA‐binding of PaeSSB, possibly preventing stable ssDNA wrapping and binding by PaeSSB^124^. Key interaction sites on PaeSSB, K7, R62, and E80, are conserved among the three SSBs of *S. aureus*, suggesting that these inhibitors could potentially apply to the SSB protein in other bacteria (Figure 6C,D). However, there are exceptions. It was reported that the SSBA inhibitor 9‐methyl‐2,3,7‐trihydroxy‐6‐fluorone (NSC5426) only weakly affects the ssDNA‐binding activity of SSBC^123^.

SSB utilizes its conserved acidic C‐terminal tails to engage with more than a dozen proteins, functioning as an organizer/mobilizer for genome maintenance^135^. High‐throughput screening campaigns have successfully identified inhibitors of SSB‐protein interactions in *E. coli* and *K. pneumoniae*, yielding numerous hits^125^, ^126^. Further validation is required to determine the applicability of these protein‐protein interaction inhibitors to SaSSBs.

Unlike EcoSSB, none of the expression of SaSSBs has been reported to be regulated by LexA in *S. aureus* ^22^. The deletion of the *S. aureus* SSBs homologous protein in *S. pneumoniae* blocks the uptake of DNA encoding the streptomycin‐resistance gene, indicating the crucial role of this protein in drug‐resistance mechanisms^136^.

*S. aureus* possesses two RecQ homologs, annotated as RecQ1 and RecQ2 (also named RecS in the KEGG database). Canonical RecQ helicases share a similar domain architecture that includes two helicase domains (HD1 and HD2), a RecQ C‐terminal (RQC) domain comprised of Zn^2+^‐binding motif, a winged‐helix (WH) domain, and a helicase and RNase D C‐terminal (HRDC) domain^137^. *S. aureus* NCTC8325 RecQ1 (SaoRecQ1) aligns well with *E. coli* RecQ (identity >33%), while *S. aureus* NCTC8325 RecQ2 (SaoRecQ2) contains a conserved N‐terminal catalytic core but lacks the WH and HDRC domains in the C‐terminal region (Figure 6E). A SaoRecQ1 missense mutation has been reported to increase the frequency of short‐sequence recombination, potentially contributing to the rapid acquisition of linezolid resistance in *S. aureus* ^138^.

Furthermore, *S. aureus* harbors RecFOR homologous proteins with high sequence identities to *E. coli* RecFOR. However, their in vivo functions and biochemical activities remain unexplored. There are no reports indicating the induction of these proteins by the SOS system in *S. aureus*. Further investigation is required to determine whether the proteins of this system in *S. aureus* are directly associated with the SOS response.

#### Other proteins that might also be related to SOS response induction

Ledger et al. utilized a reporter plasmid containing the *recA* promoter located upstream of *gfp* to monitor the induction of the SOS response^139^. From a collection of 54 mutants in the Network on Antimicrobial Resistance in *S. aureus* N315 (NARSA) transposon library, where transposons were inserted into genes previously known or proposed to be involved in DNA repair (note that in cases where two genes contributed to a single protein complex, such as RexAB, only one gene was included in the screening process), the researchers identified several mutants exhibiting a significantly reduced SOS response compared to the wild‐type strain. The top nine proteins identified include RexB, XseA, XerC, SbcC, RecG, TagD, UvrC, SA2001, and TopB^139^.

Among these proteins, RexB participates in processing DSBs in conjunction with RexA, as discussed earlier. SbcC, regulated by the SOS box in *S. aureus*, plays a crucial role in the processing of DSBs, a topic that will be further explored below. XseA is a constituent subunit of exonuclease VII, a bacterial nuclease believed to be involved in DNA repair and recombination^140^. XerC has been implicated in the monomerization of plasmids, facilitating their stable segregation during bacterial cell division^141^. RecG is thought to participate in the regression of collapsed DNA replication forks^142^. TagD, annotated as the teichoic acid biosynthesis protein D, is known to participate in glycerophospholipid metabolism according to the KEGG database. UvrC is one of the subunits that compose the nucleotide excision repair endonuclease UvrABC^143^. SA2001 contains a K^+^ ion channel beta chain regulatory domain, and this domain has been reported to exhibit oxidoreductase activity^144^. TopB, also annotated as DNA topoisomerase III, is primarily responsible for relaxing negatively supercoiled DNA and is crucial for genome maintenance^145^.

### Proteins that are regulated by LexA in *S. aureus*

**Figure 7:**
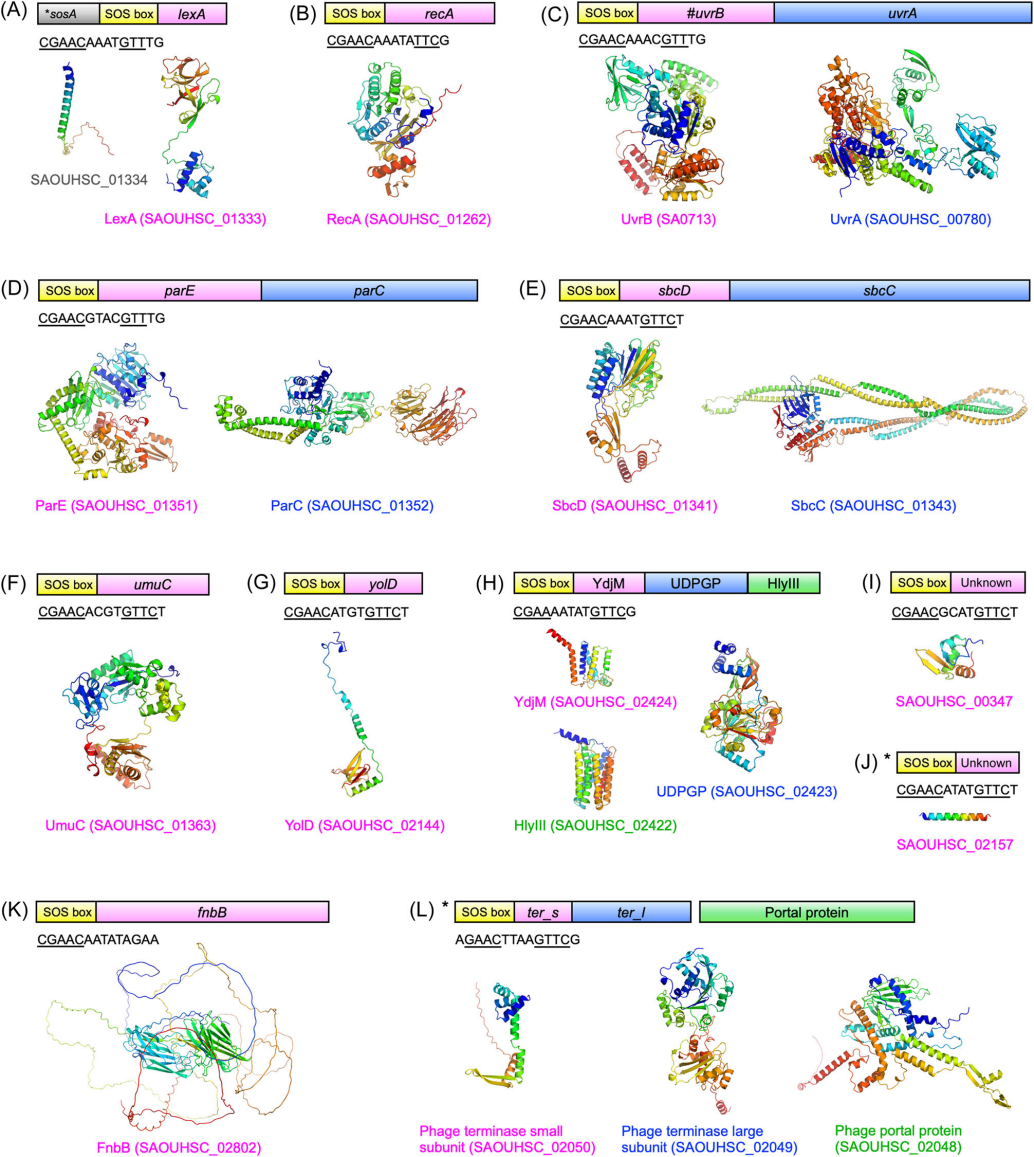
Predicted structures of proteins that are regulated by LexA in *S. aureus*. (A‐K) The schematic of the LexA regulon arrangements of *lexA* and *sosA* (A), *recA* (B), *uvrB* and *uvrA* (C), *parE* and *parC* (D), *sbcD* and *sbcC* (E), *umuC* (F), *yolD* (G), genes encoding YdjM, UDPGP, and HlyIII (H), two unknown putative genes (I, J), and *fnbB* (K) in *S. aureus* NCTC8325. (L) The schematic of the LexA regulon arrangement of genes encoding phage terminase small subunit, terminase large subunit, and portal protein in SaPI. The SOS boxes are highlighted in yellow, with the related sequence displayed below (with underlined segments denoting high conservation). Each encoded (putative) protein structure was predicted using AlphaFold2 and labeled with the protein name and its corresponding KEGG gene ID below. *this gene/operon only appears in certain *S. aureus* subspecies. # this gene is inserted by an insertion sequence (IS) fragment in *S. aureus* NCTC8325.

In *E. coli*, the regulatory control governed by LexA encompasses a substantial number of genes within the SOS regulon, consisting of at least 43 genes^133^. In contrast, Cirz et al. conducted a comparative study of the ciprofloxacin‐induced transcriptional response between the *S. aureus* LexA autoproteolysis defect mutant (S130A) strain and the wild‐type strain, identifying 18 genes induced by ciprofloxacin in the wild‐type strain but not in the mutant strain^22^. Further analysis unveiled that at least 16 genes were controlled by LexA through 10 potential SOS boxes^22^. Among the proteins regulated by LexA, both the repressor LexA and the inducer RecA of the SOS response are included, characterized by the SOS boxes “CGAACAAATGTTTG” and “CGAACAAATATTCG”, respectively (Figure 7A,B). Furthermore, numerous genes encoding proteins implicated in DNA metabolism are included in the LexA regulon, including *uvrA*, *uvrB*, *parC*, *parE*, *sbcC*, *sbcD*, *umuC*, and *yolD*. Furthermore, six genes unrelated to DNA metabolism were also found to be regulated by LexA. Although not identified in Cirz et al.'s study, the transcription of the *fnbB* gene has been found to be controlled by LexA as well^55^. In certain subspecies of *S. aureus*, particularly those genes situated on the MGEs, are also regulated by LexA. For instance, SaPI operon I, essential for SaPI packaging, is also under the control of LexA^146^. While RecA and LexA have been extensively discussed earlier, the remaining proteins will be discussed below.

#### UvrA and UvrB

The *uvrA* and *uvrB* genes encode two subunits that form the nucleotide excision repair endonuclease UvrABC^143^. UvrA interacts with UvrB to recognize damaged sites and unwind the surrounding DNA. Additionally, UvrB plays a pivotal role in recruiting other repair proteins to the damaged site, facilitating subsequent steps in the nucleotide excision repair (NER) pathway^147^. In representative strains of methicillin‐resistant *S. aureus* (MRSA), like *S. aureus* N315, and *S. aureus* USA300 strains, *uvrA* and *uvrB* are part of a single transcript regulated by the *uvrB* promoter. The LexA binding sequence for this regulation is “CGAACAAACGTTTG”^22^ (Figure 7C). Interestingly, according to the KEGG genome database, in the nonresistant strain *S. aureus* NCTC8325, a fragment of the IS5 family transposase (SAOUHSC_00777) is inserted between the internal regions of the *uvrB* gene (N‐terminal SAOUHSC_00776 and C‐terminal SAOUHSC_00779). It remains unclear whether this inserted fragment affects the downstream expression of UvrA or alters the functionality of UvrABC. If affected, this raises a question about whether the nonresistant strain's SOS response capability might be weaker compared to the resistant strains, potentially making it more sensitive to antibiotics.

The compound 2‐[5‐(methylamino)−1,3,4‐thiadiazol‐2‐yl]−3H‐benzo[f]chromen‐3‐one (ATBC) was identified as an inhibitor for NER in both *Mycobacterium* and *E. coli*. This inhibition is likely achieved by reducing the ATPase activity of UvrA^127^. The impact of ATBC on bacterial growth is observable only when it is combined with drugs that induce DNA damage, indicating its role as a cell‐penetrant, selective inhibitor^127^.

#### ParC and ParE

Topoisomerase IV is an essential enzyme involved in controlling DNA topology during DNA replication in bacteria^148^. It comprises two subunits: ParC (subunit A) responsible for DNA binding and cleavage, and ParE (subunit B), possessing ATPase activity crucial for DNA supercoiling and resealing^148^. In *S. aureus*, *parC* and *parE* are co‐transcribed and regulated by the *parE* promoter, featuring a LexA binding sequence of “CGAACGTACGTTTG” (Figure 7D). *S. aureus* also harbors DNA gyrase, consisting of GyrA and GyrB subunits, with overlapping activity spectrum with topoisomerase IV. However, unlike topoisomerase IV, DNA gyrase is not regulated by LexA in *S. aureus* ^22^.

Fluoroquinolone antibiotics primarily target both topoisomerase IV and DNA gyrase. Similar to other topoisomerase poisons, fluoroquinolones situated at the interface of the ParC and ParE subunits, stabilize a cleavage complex by trapping topoisomerase IV between the base pairs flanking the cleavage sites^128^. It has been observed that fluoroquinolones can enhance the bacterial SOS response, resulting in elevated mutation rates and the development of antibiotic resistance in persister cells^149^. Resistance to fluoroquinolones commonly arises due to mutations in ParC/GyrA, disrupting essential interactions between ciprofloxacin and the ParC/GyrA subunit. Consequently, modifications to the core structure of fluoroquinolones have been devised to counteract these forms of antibiotic resistance^150^.

Researchers have made significant progress in the development of Novel Bacterial Topoisomerase Inhibitors (NBTIs). These compounds, similar to fluoroquinolones, function as DNA intercalating agents, albeit with a focus on distinct sites. This approach allows them to circumvent specific fluoroquinolone resistance mechanisms^129^, ^130^. Recently, a 2.8 Å X‐ray crystal structure of *S. aureus* GyrA, complexed with zoliflodacin, a spiropyrimidinetrione antibiotic, unveiled the binding of this molecule to the same DNA cleavage sites as quinolones. This interaction impeded DNA re‐ligation through steric hindrance^151^. However, the effects of zoliflodacin on ParC are yet to be fully elucidated. Furthermore, extensive research has focused on inhibiting alternative binding sites of topoisomerase IV, including the second catalytic domain within topoisomerase IV and the ATPase domain found in ParE, as reviewed elsewhere^152^.

Several natural products have been previously identified, offering alternative modes of inhibiting the activity of topoisomerase IV. Kibdelomycin (KBD), a rare and recently discovered natural product antibiotic, inhibits bacterial gyrase and topoisomerase IV^131^. Notably, KBD demonstrates a low frequency of drug resistance and exhibits no cross‐resistance to *S. aureus* strains resistant to other known gyrase inhibitors^131^. The reported structure of a 43 kDa fragment of *S. aureus* ParE in complex with KBD (PDB ID: 4URL) revealed a unique U‐shaped, multicontact binding mode of KBD, distinguishing it from the binding modes of other known gyrase inhibitors, such as coumarins and quinolones. This distinctive binding mode elucidates its lack of cross‐resistance and low resistance incidence^153^.

The Fic (filamentation induced by cyclic AMP) domain is a widely distributed motif that regulates cellular activities by facilitating the transfer of the AMP moiety from ATP to protein substrates. Research has demonstrated that Fic‐2 and Fic‐1 proteins AMPylate ParE, subsequently inhibiting its activity. This inhibition leads to cell filamentation, resembling the characteristic phenotype of *parEC* deletion, wherein nucleoids gather in the center of elongated cells, a process that is accompanied by the induction of the SOS response^132^.

#### SbcC and SbcD

The SbcCD endonuclease, composed of the SbcD nuclease and the SbcC ATPase, plays a crucial role in processing DSBs in bacteria. The eukaryotic Mre11‐Rad50 complex serves as both a functional and structural homolog of the SbcCD complex^154^. In *S. aureus*, *sbcC* and *sbcD* genes are located in the same operon, which is regulated by the *sbcD* promoter. The LexA binding sequence for this regulation is “CGAACAAATGTTCT”^22^ (Figure 7E).

The crystal structure of the *E. coli* SbcCD in complex with its substrate has been determined (PDB ID: 6S85)^155^. Structural and biochemical studies of *S. aureus* SbcD have demonstrated a dimeric assembly and endonuclease activity akin to that of *E. coli* SbcD^156^. Notably, unlike in *S. aureus*, the regulation of SbcCD by LexA does not occur in *E. coli* ^157^.

#### UmuC and YolD

In *E. coli*, Pol V is constituted by the trimeric complex UmuD′2C, encoded by the *umuDC* operon. Initially, full‐length *E. coli* UmuD (EcoUmuD) remains inactive. However, upon induction of the SOS response, the activated RecA‐ssDNA nucleoprotein filament facilitates self‐cleavage of UmuD to its active form, UmuD′. Subsequently, UmuD′ complexes with UmuC, serving as an error‐prone DNA polymerase that allows replication across damaged regions, albeit with elevated error rates^158^. Interestingly, studies have revealed that UmuC contributes to the survival of *S. aureus* during amino acid starvation, a factor that activates the SOS response^159^.

The catalytic subunit of Pol V, UmuC, belong to the Y‐family, specializing in translesion DNA synthesis across diverse DNA adducts to restore stalled chromosomal replication at the cost of inducing mutations^160^. In *S. aureus*, the expression of the UmuC subunit is repressed by LexA through the SOS box “CGAACACGTGTTCT” (Figure 7F). However, *S. aureus* lacks an *umuD* homolog. Instead, it contains a *yolD* gene, whose expression is regulated by LexA using the SOS box “CGAACATGTGTTCT”^22^ (Figure 7G). It has been suggested that YolD in *S. aureus* carries out the function of UmuD^22^, ^161^. Despite lacking sequence identity between UmuD and YolD, they exhibit somewhat similar conformations. The Alphafold2‐predicted *S. aureus* YolD comprises a flexible NTD and a C‐terminal OB‐fold‐like domain, similar to EcoUmuD, which also includes a C‐terminal OB‐fold‐like domain^162^. However, without experimental evidence, it remains unclear whether YolD collaborates with UmuC.

The self‐cleavage site of EcoUmuD is situated between C24 and G25. Following cleavage, UmuD′ loses 24 N‐terminal amino acids and forms a complex with UmuC^163^. In the crystal structure of EcoUmuD′, the catalytically active S60 and K97 residues of the EcoUmuD′ enzyme are positioned at the end of a cleft within the protein's globular body, ready for cleavage^162^. However, YolD lacks corresponding cleavage sites and catalytic residues, suggesting it may employ different mechanisms from UmuD.

No specific inhibitors targeting Pol V or YolD have been reported to date.

Moreover, in *E. coli*, SOS‐induced error‐prone DNA repair is facilitated by polymerase B (PolB or Pol II), DinB, or UmuD′2C^164^. However, neither PolB nor DinB has been proven to be regulated by the SOS response in *S. aureus* ^22^.

#### Other LexA‐regulated genes in *S. aureus*

In *S. aureus*, there exists a polycistronic operon, regulated by LexA, encompassing three uncharacterized genes. These genes encode a protein containing the YdjM motif, a protein containing the UDPGP motif, and a protein containing the HlyIII motif^22^ (Figure 7H). The SOS box for this operon is “CGAAAATATGTTCG”. In *E. coli*, the homologous protein containing the YdjM motif is also regulated by LexA and is predicted to function as a membrane‐bound, metal‐dependent hydrolase, possibly acting as a phospholipase^165^. The protein containing the HlyIII motif is a putative hemolysin. Notably, in *E. coli*, the gene encoding the homologous protein with the HlyIII motif is not located within the same operon as the gene encoding the homologous protein with the YdjM motif and is not regulated by LexA^165^. The protein containing the UDPGP motif is annotated as a putative uridylyltransferase in the KEGG database. No highly sequence‐identical homologous protein has been identified in *E. coli*.

In certain *S. aureus* subspecies, such as *S. aureus* NCTC8325 and *S. aureus* N315, a Sur_N motif‐containing protein, annotated as SosA, is located upstream of LexA and is presumed to be regulated by LexA^22^. SosA, a concise 77‐amino‐acid protein, lacks conservation among staphylococci^64^. Its AlphaFold2‐predicted structure comprises an N‐terminal transmembrane helix and a C‐terminal extracellularly located loop (Figure 7A). SosA can only be derepressed following the further digestion of its NTD by Clp proteases, as previously mentioned^74^, ^75^. Despite the absence of significant sequence similarity among LexA‐regulated *S. aureus* SosA, *E. coli* SulA, and *B. subtilis* YneA, they all share the common function of inhibiting cell division to aid in DNA damage repair^64^.

Additionally, two putative standalone uncharacterized genes are regulated by LexA^22^. One of these genes is a putative 192‐base gene with an SOS box “CGAACGCATGTTCT” (Figure 7I). Its upstream gene encodes a ribosome‐binding ATPase, while its downstream genes encode a 30S ribosomal protein S6, an SSBA protein, and a 30S ribosomal protein S18. Interestingly, while the expression of SSBA in *S. aureus* appears not to be governed by LexA, its homolog is indeed regulated by LexA in *E. coli* ^157^. The other is a putative 99‐base gene (SAOUHSC_02157) with the same SOS box sequence, “CGAACATATGTTCT” (Figure 7J). It remains uncertain whether these short DNA sequences encode functional proteins or peptides. *E. coli* lacks homologous genes for both of these putative genes.

The transcription of the *fnbB* gene has been found to be controlled by SaLexA through the SOS box “C GAACAATATAGAA”^55^. *fnbB* encodes the fibronectin‐binding protein B (FnbB), which is crucial for accelerating biofilm formation (Figure 7K)^55^. The SOS box of *fnbB* is not a canonical *S. aureus* SOS box, as only four bases are conserved. Based on this SOS box, other putative *S. aureus* SOS boxes were discovered in the promoter regions of the *recQ1* (“C GAACGATTAAAAT”), *recN* (“C GAAGCAAAGAGGC”), and *uvrC* (“C GAAGATGTTGATT”) genes in the same study^55^. Further confirmation is needed to determine whether the transcription of *recQ1*, *recN*, and *uvrC* is regulated by LexA in *S. aureus*.

Furthermore, the SaPIbov1 operon I, located on the prophage in specific subspecies of *S. aureus*, crucial for SaPI packaging, has been reported to be under the control of LexA. The SOS box sequence of SaPI operon I was identified as “AGAACTTAAGTTCG”^146^. This operon encodes a homolog of the phage terminase small subunit, plus two other genes that direct the package of SaPI DNA and assembly of the SaPI‐specific small capsids (Figure 7L).

## PERSPECTIVES OF ANTIBIOTICS TARGETING *S. AUREUS* SOS RESPONSE

Previously, efforts to address resistance have primarily concentrated on modifying existing antibiotics to bypass the molecular mechanisms that confer resistance^166^. While these endeavors have proven effective against resistant strains, new resistance mechanisms often emerge during the adaptation to new antimicrobial agents^167^. Several studies have investigated various aspects of bacterial DNA repair as potential targets for exploring and developing novel antibiotics^97^. Since antibiotic resistance is usually acquired by activating bacterial SOS‐dependent mutagenesis and horizontal gene transfer pathways, compounds capable of inhibiting the SOS response are crucial for devising new combinational strategies to block mutagenesis^168^. Additionally, they could be used alongside ciprofloxacin or other conventional antibiotics to combat invasive infections and restore bacterial susceptibility to ciprofloxacin, presenting a novel therapeutic approach^169^.

Traditional broad‐spectrum antibiotics targeting cell walls, cell membranes, and molecular synthesis/metabolism might not discriminate between pathogenic and beneficial bacteria due to the conserved nature of the drug targets, leading to multiple side effects during therapy. It is important to note that another significant advantage of targeting the SOS response lies in the fact that these inhibitors are not essential for bacterial growth and only become crucial in the event of DNA damage^1^. Consequently, during an infection, SOS response inhibitors are expected to have the most substantial impact on the targeted pathogenic bacteria under assault from the immune system. This suggests that adverse effects on the commensal gut microbiota would be minimized compared to conventional antibiotic treatments^170^.

In addition to inhibitors targeting specific SOS response proteins mentioned above, there are also compounds known to inhibit the global regulation of the SOS response in *S. aureus*. For instance, a study by Peng et al.^171^ revealed that baicalein, a primary component of the Chinese herb *Scutellaria baicalensis*, effectively inhibited the expression of key SOS genes (*recA*, *lexA*, and *umuC*) in *S. aureus*. This inhibitory effect was associated with a decrease in intracellular ATP production, indicating that baicalein might target ATP synthase. A study in *S. aureus* indicated that juglone, a natural naphthoquinone, not only exhibited antibiotic activity by inducing ROS production in bacterial cells but also reduced bacterial DNA repair by inhibiting RecA expression^172^. The DNA gyrase inhibitor novobiocin also demonstrated the ability to suppress the ciprofloxacin‐induced SOS response in *S. aureus* by impeding RecA expression^59^. Betulinic acid, a triterpenoid derived from plants, has also shown SOS‐inhibiting properties by reducing the ciprofloxacin‐induced activation of the SOS response and enhancing ciprofloxacin's activity in *S. aureus* ^173^.

However, currently, only a very limited number of drugs targeting the SOS response process mentioned above have been employed in clinical trials, and some have been confirmed to have notable drawbacks. Although fluoroquinolone antibiotics can inhibit the activity of SOS response effector protein ParEC in *S. aureus*, causing double‐strand breaks (DSBs) and leading to bacterial death, this effect can, in turn, trigger the SOS response, enhancing the emergence of mutants and drug‐resistant strains^149^. Polysulfated naphthyl compounds, reported as RecA inhibitors, are likely to have limited therapeutic utility due to their negative charges causing membrane impermeability^90^. Also, as mentioned earlier, inhibitors targeting RecA may present potential host toxicity due to the presence of bacterial RecA homologous proteins in host cells^99^.

In addition to new drugs, there are also alternative strategies targeting the bacterial SOS response. The llama‐derived nanobodies, specifically engineered to target LexA autoproteolysis, present a viable option for selectively combating bacterial infections^81^.

Phages show promise as tools to combat antibiotic‐resistant bacteria. Combined treatments involving phages and various antibiotics result in enhanced bacterial eradication, known as phage‐antibiotic synergy. When exposed to sublethal doses of antibiotics, the activation of the SOS response results in the formation of filamentous cells that halt division, rendering them vulnerable to targeting and lysis by phages^174^. Moreover, while phages are often implicated as carriers that facilitate the transfer of pathogenic and antibiotic resistance genes during the SOS response, potentially leading to adverse effects in clinical antimicrobial therapy, certain phages harbor proteins capable of inhibiting the SOS response. Examples include the inhibitory protein phage GIL01 gp7, which hinders the autocleavage of LexA, as well as Lambda Gam, T7 gp5.9, and P22 Abc2, which inhibit the digestion activity of RecBCD. The presence of these inhibitory proteins can help alleviate the aforementioned adverse effects.

In summary, the accumulating evidence underscores the vital role that the SOS response system plays in the pathogenicity of *S. aureus*. Inhibitors directed at the SOS response system aim to bolster the pathogen's vulnerability to both host immune responses and antibiotics, concurrently mitigating the emergence of drug‐resistant and host‐adapted strains. Our deepened understanding of SOS response‐associated proteins in this bacterium actively shapes the development of next‐generation anti‐staphylococcal therapies.
